# Developing the concept of maternal in teenage mothers: a hybrid model

**DOI:** 10.3389/fpsyg.2023.1246882

**Published:** 2024-01-08

**Authors:** Akram Sadat Sadat Hoseini, Maryam Maleki, Abbas Mardani, Soheila Abbasi

**Affiliations:** ^1^School of Nursing and Midwifery and Member of Research Center of Quran, Hadith and Medicine of Tehran University of Medical Sciences, Tehran, Iran; ^2^Pediatric and Neonatal Intensive Care Nursing Education Department, School of Nursing and Midwifery, Tehran University of Medical Sciences, Tehran, Iran; ^3^Department of Medical-Surgical Nursing, School of Nursing and Midwifery, Zanjan University of Medical Sciences, Zanjan, Iran

**Keywords:** motherhood concept, maternal care, adolescent, teenage, hybrid study

## Abstract

**Objectives:**

Maternal transition is a challenging developmental process requiring physical, mental, social, and cognitive preparedness. Therefore, the transition to motherhood is even more challenging for teenagers who are exposed to both the maturation process and adaptation to the parenting role. Therefore, the present study was conducted to provide a deeper understanding of the maternal role in Iranian teenage mothers.

**Methods:**

A three-phase hybrid model was adopted for concept analysis. In the theoretical phase, 50 articles were analyzed. In the fieldwork phase, 10 participants were interviewed. In the final phase, the findings of the previous two stages were analyzed. This study was conducted between October 2021 and November 2022. After determining the attributes, antecedents, and consequences, a final definition was presented for parenting in teenage mothers.

**Results:**

The concept of motherhood for teenage mothers was described as “a process-oriented phenomenon in nature,” “complex, challenging and multidimensional,” “development with immature transition,” “a turning point in life,” “a stressful event,” “affected by several factors,” and “bidirectional effects on life” according to antecedents, including “the level of received social support,” “reaction to teenage motherhood,” “teenage mother’s characteristics,” and “family structure” leading to “positive psychological consequences,” “negative psychological consequences,” and “loss of educational and career opportunities.”

**Conclusion:**

This study provides a suitable ground for evaluating the concept of motherhood in teenage mothers and employing it in nursing care of teenage mothers and children by identifying this concept.

## 1 Introduction

Motherhood is an important role for women in all societies (Javadifar et al., 2016). Motherhood is a transition from a current known situation to a new, unknown reality (DeVito, 2010). In the process of becoming a mother, a period of change, instability, and reorganization is experienced (Javadifar et al., 2016), which begins before the birth of the baby and continues until after birth. Maternal transition requires physical, mental, social, and cognitive preparedness. Therefore, the mother needs capabilities to accomplish this new situation (Aparicio et al., 2015).

Adolescence is the time when a person tries to build adult capabilities (DeVito, 2010); therefore, the maternal transition is challenging for teenagers who are going through the puberty process and coping with the maternal role at the same time (WHO, 2020). In one study, teenage African-American mothers described becoming a mother as a feeling of “being caught between two worlds,” indicating the challenges of a teenage mother between her social and parenting needs and the needs related to her stage of adolescent development (DeVito, 2010).

Communication and friendship with peers are important in adolescence and create a feeling of social acceptance and stability in teenagers. Teenage mothers usually experience problems in maintaining their friendships and social activities with their former friends who have no perception of the needs of an adolescent parent; therefore, they usually feel lonely and abandoned. Adolescents need to sleep more than adults; therefore, a teenage mother may experience more sleep deprivation as a result of constant infant care compared to an adult mother (DeVito, 2010). Teenage mothers are at a high risk of puerperal endometritis, eclampsia, systemic infections, preterm delivery, low birth weight, and severe diseases of the newborn compared to their adult counterparts (Gbogbo, 2020). In most cases, poverty is added to the many problems and challenges of teenage mothers, which can affect their and their infants’ health (Kagawa et al., 2017).

Poor capabilities of teenage mothers for family management, their limited experience in infant care, and their emotional ups and downs result in a feeling of incompetence and lack of self-confidence and hinder their maternal role (Kagawa et al., 2017). As a result of becoming a mother, they usually lose their educational and consequently occupational opportunities and feel hopeless (Bah, 2016). These problems drive many teenage mothers toward unpleasant feelings like unhappiness, shock, low self-confidence, and confusion about motherhood (Pitso and Kheswa, 2014). Hence, the meaning of motherhood for teenagers may cover a spectrum of feelings from positive ones like the transition to adulthood and development through becoming a mother to negative ones like accepting the burden of infant care before mental and physical preparedness (Bah, 2016). For example, stressful events in the everyday life of teenage African-American mothers did not prevent them from loving their babies and they defined the concept of parenting with phrases such as mothering under pressure, a fragile network, sharing responsibilities, building a network, and seeing the future (Dole and Shambley-Ebron, 2016). In a study using qualitative content analysis, when adolescent African-American mothers were asked to express their feelings and needs related to parenting and caring for their infants, they stated that they were unable to answer and share their feelings and thoughts (DeVito, 2010). In addition, the lived experiences of adolescent Mexican-American mothers about the concept of motherhood included ethnic and racial discrimination, strong attachment toward the infant, an opportunity to preserve their traditional familial cultural heritage, and an attempt to create a nostalgic sense of motherhood (Sommer et al., 2019). Adolescent Belgian mothers defined becoming a mother with phrases such as a feeling of being valued, happiness, pride, maturity, and responsibility and considered the child as a support (Aujoulat et al., 2010). Adolescent British girls who became mothers in adolescence had a positive attitude and valuable feelings toward becoming a mother and child-rearing. Some of the mothers viewed mothering as a motive for changing their path and considering an occupation since they believed that they were responsible for another person. They also stated that becoming a mother in adolescence did not mean that their life and future were all over. Most of them were realistic about their future and planned career development options (Seamark and Lings, 2004).

### 1.1 Context of Iran

The practice of early marriage is deeply ingrained in Iranian society (Meghdadi and Javadpour, 2017). According to Article 1041 of the Civil Law, girls as young as 13 years old are legally allowed to marry. In exceptional cases, such as when the girl’s parents or the court deem her ready for marriage, girls under the age of 13 may also be married (Tremayne, 2006). In Iran, almost a quarter of registered marriages involve women under the age of 19 (Ahmady, 2021). In addition, the Iranian Government’s Strategy Plan includes significant investments to increase the population rate in the next decade. As a result, it is anticipated that the fertility rate among young Iranians will notably rise by the next decade. Since Iranian girls tend to become pregnant shortly after marriage, they are confronted with multiple life-changing events, including marriage, pregnancy, and motherhood, during their transition into adulthood. Therefore, the issue of adolescent marriage, early pregnancy, and childbearing is a complex problem that affects families, healthcare providers, educators, government officials, and young people in Iran.

The diversity of the mothers’ experiences shows the effect of social and cultural factors on the concept of motherhood in adolescents. The previous research has primarily taken place in nations where teenage pregnancies may happen outside of marriage. The situation in Iran is different from theirs, as most adolescent pregnancies in Iran happen within the confines of legal marriages, resulting in a unique pregnancy experience. In addition, in Iran, teenage pregnancy following marriage is socially accepted. Furthermore, pregnancy and motherhood within marriage probably gave them more family and social support and reduce social stigma. Moreover, Iranian culture is a patriarchal culture, and women, in addition to their motherly roles, have to do most of the family related tasks such as housework, which can be challenging, especially for teenage mothers. Therefore, it seems that the concept of motherhood in Iranian teenagers is different from other cultures.

Considering the complexities and diversities of the concept of motherhood in different geographical, cultural, and social structures, it is necessary to address the concept of motherhood from different perspectives to explore this situation and improve the process of motherhood in adolescents (Atuyambe et al., 2009; Mohammadi et al., 2016). Therefore, the present study was conducted to provide a deep understanding of the concept of motherhood in Iranian teenage mothers using a hybrid model.

## 2 Materials and methods

### 2.1 Study design

A combination of literature search and qualitative research was used to conduct this study in 2022. A concept development study was conducted using a hybrid model with three stages, including the theoretical phase, fieldwork phase, and final analysis. Hybrid concept analysis can enrich the concept of adolescent motherhood through qualitative analysis of the adolescent mothers’ perspectives.

### 2.2 Theoretical analysis of the motherhood concept among teenage mothers

Theoretical analysis is the first step in hybrid concept analysis. In this phase, for a literature search, the papers published on motherhood in teenage mothers were searched in four databases including the Web of Science, PubMed, Scopus, and Embase using the keywords “adolescent mother,” “teenage motherhood,” and “becoming a mother.” The advanced search options of the databases were used to combine the keywords. The inclusion criteria were original and review articles that were published between 2000 and 2022 and focused on the concept of motherhood in teenagers. The exclusion criteria included non-English language articles and studies that did not focus on motherhood in teenagers. Two authors (MM and SA) independently conducted the review process, including selecting relevant studies. The EndNote software was utilized for data management. Following the search, all titles were reviewed, and the abstracts and then full texts of chosen studies were carefully examined and the relevant studies were included in the concept analysis. In cases where there was a disagreement regarding the inclusion of studies in the concept analysis, discussions were held with a third author (ASSH) to reach a consensus. Finally, 50 articles related to motherhood in adolescent mothers were found. The studies were evaluated using the following questions:

- How is motherhood defined by adolescent mothers?
- What is the concept of motherhood among adolescent mothers?
- How is motherhood measured among adolescent mothers?

To extract the attributes and factors related to the concept of motherhood among adolescent mothers, the articles were evaluated comprehensively and a summary was provided for each one. Each relevant text was read several times to gain a general understanding using the content analysis method (Vaismoradi et al., 2016).

Analysis of the retrieved studies ended with a definition of the concept of motherhood among teenage mothers. As a result, the maternal attributes in adolescent mothers, antecedents, and consequences were determined. This stage was completed by presenting an operational definition of motherhood in teenage mothers (Supplementary File 1).

### 2.3 Fieldwork phase

The objective of this phase was to reinforce and refine the concept developed in the first phase (Rafii et al., 2014). Qualitative content analysis was used to understand the concept better. The inclusion criteria were age under 19 years, first delivery, having an infant under 6 months of age, having a healthy infant, willingness to participate in the study, and the ability to share information and experiences. Mothers with sick infants, preterm delivery, and post-delivery mental disorders were excluded from the study. The participants were selected using purposive sampling with maximum diversity in terms of education level, age, and living place. The data were collected through semi-structured, in-depth, face-to-face interviews. Data collection continued until data saturation was reached. The interviews lasted 30–45 min on average. The questions that were asked to elucidate the concept were, “Describe your experience becoming a mother,” “How did your situation change when you became a mother?,” and “What did you learn from this experience?” In addition, according to the participants’ answers, probing questions were asked such as, “Please explain more,” “Describe your feeling at that moment,” and “Please specify what changed exactly.”

Data analysis was done using the content analysis method proposed by Graneheim and Lundman (2004). The interviews were read through line by line several times for immersion in the data and to gain a better understanding of the contents of the interviews. Then, the meaning units were identified and labeled using the open-coding process. The codes were grouped into developed categories and subcategories based on similarities and differences (Vaismoradi and Snelgrove, 2019). The key attributes of the motherhood concept among adolescent mothers were identified accordingly.

### 2.4 Final analysis phase

In this phase, the results of the previous phases were analyzed and compared. Similar codes were merged under a unique title and non-emerged codes were presented as separate codes. Finally, a comprehensive definition of motherhood in adolescent mothers was obtained, and its attributes, antecedents, and consequences were categorized. During the merging, fieldwork findings had precedence over theoretical phase findings since the aim of the study was to focus on the experiences of adolescent Iranian mothers.

### 2.5 Trustworthiness of data

Four criteria including credibility, dependability, confirmability, and transferability were used to evaluate the trustworthiness of data as proposed by Polit and Beck (2004). For this purpose, the researchers considered prolonged engagement with the participants and the research topic to gain the participants’ trust and develop a deeper understanding of the study environment. Member checking was done to ensure the accuracy of the data and codes. In other words, after coding, the transcripts of the interviews were shared with the participants to confirm the accuracy of the codes and interpretations, and the codes that did not reflect their views were corrected. Maximum variation in terms of age, occupation, culture, social class, and education level was ensured while selecting the participants. The transcripts of the interviews were evaluated by the research team and the extracted codes and categories were shared with a number of faculty members for confirmation.

### 2.6 Ethical considerations

The study protocol was approved by the Ethics Committee of Tehran University of Medical Sciences (IR.TUMS.MEDICINE.REC.1400.815). Informed consent was obtained from the participants before entering the study and the interviews were recorded with permission.

## 3 Results

### 3.1 Theoretical phase

#### 3.1.1 Concept definition

The term “motherhood” is defined as the state or time of being a mother, and the term “maternal” is an equivalent. Motherhood is defined as having an internalized view of the self as a mother (Polit and Beck, 2004).

#### 3.1.2 Concept attributes

Attributes are described as important features (signs and symptoms) of a concept that appear frequently during the analysis of the concept and help to clarify one concept and distinguish it from another interrelated concept (Nukpezah et al., 2020). Analysis showed seven categories of attributes, including “a process-oriented phenomenon in nature,” “complex, challenging and multidimensional,” “development with immature transition,” “a turning point in life,” “a stressful event,” “affected by multiple factors,” and “bidirectional effects on life.” In fact, motherhood has different aspects, including cultural, cognitive, social, legal, behavioral, and health aspects (Mangeli et al., 2017; Campbell and Hart, 2019; Nkwemu et al., 2019; Apolot et al., 2020; Chemutai et al., 2020; Govender et al., 2020; Santos et al., 2021; Twintoh et al., 2021; Wainaina et al., 2021). A review of the literature showed that becoming a mother is an unexpected and immature transition and development in adolescents (Macintosh and Callister, 2015; Margherita et al., 2017; Mangeli et al., 2018a; Dzotsi et al., 2020; Gbogbo, 2020; Gharacheh et al., 2020; Santos et al., 2021). Therefore, motherhood is a turning point in the life of a teenager that can cause a fundamental transformation (Seamark and Lings, 2004; Soares and Lopes, 2011; Moridi and Aminshokravi, 2018; Zainudin, 2019; Santos et al., 2021). As a result of major physical, mental, social, and cognitive changes during pregnancy and early motherhood, transition to motherhood is considered a stressful process with many responsibilities (Rentschler, 2003; Atuyambe et al., 2005; Soares and Lopes, 2011; James et al., 2012; Anwar and Stanistreet, 2015; Kagawa et al., 2017; Mangeli et al., 2018a; Dhaka and Musese, 2019; Diamand et al., 2019; Franco-Ramírez et al., 2020; Gharacheh et al., 2020; Govender et al., 2020; Malatji et al., 2020; Tirgari et al., 2020; Kazal et al., 2021; Naidoo et al., 2021).“Bidirectional effects on life” was another attribute of motherhood in teenage mothers, indicating that motherhood is a process of learning (Kaye, 2008; Klingberg-Allvin et al., 2008; Kagawa et al., 2017; Santos et al., 2021; Erfina et al., 2022) and an opportunity to develop, strengthen marriage, and make up for the childhood and adolescence deprivations which can create positive and meaningful paths in life. However, it is a vulnerable period (Kaye, 2008; Margherita et al., 2017; Zainudin, 2019) and a negative experience for teenage mothers, their children, and society (Klingberg-Allvin et al., 2008; Anwar and Stanistreet, 2015; Zainudin, 2019); therefore, it can be stated that teenage mothers wilt before blooming (Gharacheh et al., 2020).

#### 3.1.3 Concept antecedents

Antecedents are described as actions or measures that must occur first hand before a concept is concluded (Nukpezah et al., 2020). According to the results of the theoretical phase, the antecedents of motherhood in teenage mothers were “the level of received social support,” “reaction to teenage motherhood,” “teenage mother’s characteristics,” and “family structure.” A review of the literature showed that “the level of received social support” was one of the antecedents of motherhood in teenage mothers, which is referred to as “social support” in the literature (Kagawa et al., 2017; Mangeli et al., 2018b; Dhaka and Musese, 2019; Nkwemu et al., 2019; Field et al., 2020; Gbogbo, 2020; Gharacheh et al., 2020; Govender et al., 2020; Recto and Champion, 2020; Tirgari et al., 2020; Kazal et al., 2021; SmithBattle et al., 2021), including family members’ support (Atuyambe et al., 2005; Kaye, 2008; James et al., 2012; Ngum Chi Watts et al., 2015; Kagawa et al., 2017; Mangeli et al., 2017; Osok et al., 2018; Santos et al., 2018, 2021; Amod et al., 2019; Campbell and Hart, 2019; Diamand et al., 2019; Chemutai et al., 2020; Gbogbo, 2020; Govender et al., 2020; Malatji et al., 2020; Recto and Champion, 2020; Kazal et al., 2021; Twintoh et al., 2021; Erfina et al., 2022), spouse’s support (Atuyambe et al., 2005; Kaye, 2008; James et al., 2012; Ngum Chi Watts et al., 2015; Mangeli et al., 2017; Osok et al., 2018; Amod et al., 2019; Campbell and Hart, 2019; Franco-Ramírez et al., 2020; Gbogbo, 2020; Govender et al., 2020; Santos et al., 2021; Erfina et al., 2022), healthcare providers’ support (Mangeli et al., 2017, 2018b; Campbell and Hart, 2019; Recto and Champion, 2020), and government’s support (Atuyambe et al., 2005; Kaye, 2008; Klingberg-Allvin et al., 2008; Mangeli et al., 2018b; Amod et al., 2019; Dhaka and Musese, 2019; Naidoo et al., 2021). Another antecedent of motherhood in teenage mothers was the reaction of the family, friends, teachers, healthcare providers, and society to parenting as a teenager with the most common reaction being stigma (Sámano et al., 2017; Moridi and Aminshokravi, 2018; Osok et al., 2018; Sychareun et al., 2018; Amod et al., 2019; Campbell and Hart, 2019; Dhaka and Musese, 2019; Nkwemu et al., 2019; Apolot et al., 2020; Calver, 2020; Chemutai et al., 2020; Dzotsi et al., 2020; Field et al., 2020; Gbogbo, 2020; Gharacheh et al., 2020; Govender et al., 2020; Malatji et al., 2020; Recto and Champion, 2020; Naidoo et al., 2021; SmithBattle et al., 2021; Wainaina et al., 2021). Another antecedent of the concept of motherhood was the “teenage mother’s characteristics.” The subcategories that formed this category were the pregnancy status (Margherita et al., 2017; Sychareun et al., 2018; Amod et al., 2019; Chemutai et al., 2020; Franco-Ramírez et al., 2020; Gbogbo, 2020; Govender et al., 2020; Tirgari et al., 2020), education level (Klingberg-Allvin et al., 2008; Amod et al., 2019; Field et al., 2020; Gbogbo, 2020; Tirgari et al., 2020; Kazal et al., 2021; Twintoh et al., 2021), and physical and skill capabilities (Mangeli et al., 2017, 2018a; Moridi and Aminshokravi, 2018; Osok et al., 2018; Erfina et al., 2019; Apolot et al., 2020; Gharacheh et al., 2020; Tirgari et al., 2020; Kazal et al., 2021; Twintoh et al., 2021). “Family structure” included the economic state (Atuyambe et al., 2005; Kagawa et al., 2017; Mangeli et al., 2017; Osok et al., 2018; Santos et al., 2018; Amod et al., 2019; Dhaka and Musese, 2019; Moridi et al., 2019; Nkwemu et al., 2019; Chemutai et al., 2020; Dzotsi et al., 2020; Gbogbo, 2020; Govender et al., 2020; Recto and Champion, 2020; Twintoh et al., 2021; Wainaina et al., 2021) and culture and traditions (Dehghan-Nayeri and Tajvidi, 2014; Mangeli et al., 2017; Sámano et al., 2017; Osok et al., 2018; Sychareun et al., 2018; Amod et al., 2019; Campbell and Hart, 2019; Dhaka and Musese, 2019; Nkwemu et al., 2019; Sommer et al., 2019).

#### 3.1.4 Concept consequences

“Negative psychological consequences” included a feeling of humiliation, shame, fear, stress, anxiety, depression, shock, guilt, anger, suicide thoughts, isolation, and identity confusion (Rentschler, 2003; Atuyambe et al., 2005; Kaye, 2008; DeVito, 2010; Soares and Lopes, 2011; James et al., 2012; Mangeli et al., 2017; Margherita et al., 2017; Sámano et al., 2017; Osok et al., 2018; Santos et al., 2018, 2021; Amod et al., 2019; Campbell and Hart, 2019; Dhaka and Musese, 2019; Diamand et al., 2019; Moridi et al., 2019; Nkwemu et al., 2019; Sommer et al., 2019; Apolot et al., 2020; Calver, 2020; Chemutai et al., 2020; Franco-Ramírez et al., 2020; Gbogbo, 2020; Gharacheh et al., 2020; Govender et al., 2020; Malatji et al., 2020; Recto and Champion, 2020; Tirgari et al., 2020; Kazal et al., 2021; Naidoo et al., 2021; Wainaina et al., 2021).“Positive psychological consequences” included gaining respect (Anwar and Stanistreet, 2015; Mangeli et al., 2017; Calver, 2020; Gharacheh et al., 2020), feelings of joy (Klingberg-Allvin et al., 2008; Moridi et al., 2019; Sommer et al., 2019; Chemutai et al., 2020; Franco-Ramírez et al., 2020), maturity, reduced risky behavior (Anwar and Stanistreet, 2015; Macintosh and Callister, 2015; Ngum Chi Watts et al., 2015; SmithBattle et al., 2021), learning (Dhaka and Musese, 2019; Santos et al., 2021; Erfina et al., 2022), an opportunity for positive changes (Seamark and Lings, 2004; Ngum Chi Watts et al., 2015; Diamand et al., 2019), acquiring motherhood competencies (Seamark and Lings, 2004; Macintosh and Callister, 2015; Ngum Chi Watts et al., 2015; Mangeli et al., 2018b), closeness to God, spiritual empowerment (Zainudin, 2019), enjoying a sense of independence and self-confidence, and improvement in marital life (Seamark and Lings, 2004; Klingberg-Allvin et al., 2008; Aparicio et al., 2015; Moridi and Aminshokravi, 2018). The third consequence was losing career and educational opportunities (Rentschler, 2003; Atuyambe et al., 2005; Soares and Lopes, 2011; Anwar and Stanistreet, 2015; Kagawa et al., 2017; Margherita et al., 2017; Mangeli et al., 2018a; Osok et al., 2018; Santos et al., 2018, 2021; Dhaka and Musese, 2019; Nkwemu et al., 2019; Sommer et al., 2019; Dzotsi et al., 2020; Franco-Ramírez et al., 2020; Gbogbo, 2020; Gharacheh et al., 2020; Govender et al., 2020; Tirgari et al., 2020; Wainaina et al., 2021).

#### 3.1.5 Operational definition of concept based on literature review

**FIGURE 1:**
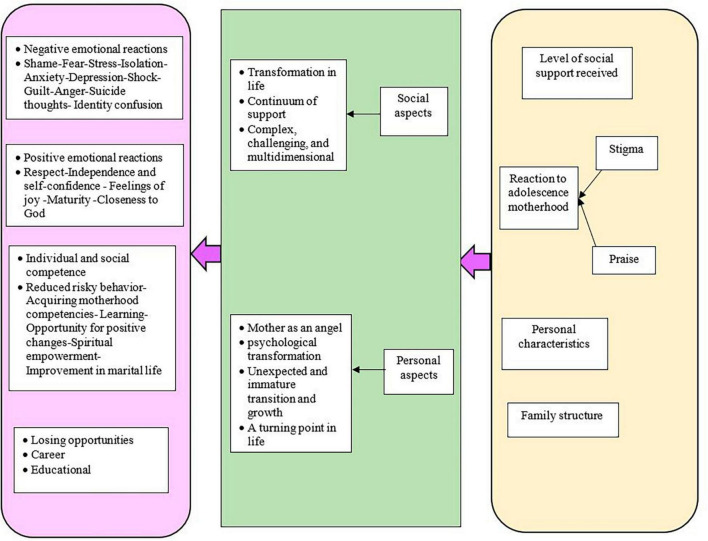
Theoretical phase findings.

Based on the literature review, operationally, motherhood in adolescence is considered a process that is challenging, evolving, multidimensional, and complex. It involves the level of received social support, reaction to adolescent motherhood, attributes of the adolescent mother, and family structure leading to positive and negative psychological consequences and loss of educational and occupational opportunities (Figure 1).

### 3.2 Fieldwork phase

The participants included 10 adolescent mothers aged 16 to 18. The demographic characteristics of the participants are presented in Table 1. In this stage, after completing the qualitative content analysis of the interviews with mothers, initial codes were extracted, which were classified into 5 categories and 16 subcategories (Table 2). The categories were “mother as an angel,” “transition in life,” “continuum of support,” “emotional reactions to pregnancy and becoming a mother in adolescence,” and “adolescent mother vs. adult mother.”

**TABLE 1:** Demographic characteristics of participants.

Participant	Maternal age (year)	Child’s age (months)	Education level (grade)	Economic state
1	18	15	10th	Poor
2	18	9	8th	Poor
3	18	12	9th	Poor
4	18	24	9th	Poor
5	19	7	9th	Intermediate
6	18	5	9th	Poor
7	16	6	9th	Poor
8	17	9	9th	Poor
9	18	18	9th	Poor
10	18	18	9th	Poor

**TABLE 2:** Fieldwork phase findings.

Categories	Sub-categories
Mother as an angel	Responsibility and patience in hardships
	Love and kindness
	Selflessness and sacrifice
	Caretaker and sanctuary
Transformation in life	Psychological transformation
	Diversity and enthusiasm
	Changes in relationships
	Losses
	Experienced personal physical problems
Continuum of support	Perceived sufficient support
	Insufficient support
Emotional reactions to pregnancy and becoming a mother in adolescence	Positive emotional reactions
	Negative emotional reactions
Adolescent mother vs. adult mother	Better understanding of child by adolescent mother
	Greater enthusiasm of adolescent mother
	Higher experience of adult mother

#### 3.2.1 Mother as an angel

This category described the attributes of motherhood from the perspective of adolescent mothers. Adolescent mothers viewed motherhood as an angelic role with unique attributes. These attributes were classified into four subcategories: “responsibility and patience in hardships,” “love and kindness,” “selflessness and sacrifice,” and “caretaker and sanctuary.”

One of the participants said, “*Being a mother means enduring a lot of hardships. Now I understand why they say heaven is at a mother’s feet. Because it’s truly only a mother who can go through all this difficulty for someone’s sake*” (Participant 1).

Most participants depicted mothers as self-sacrificing and caring individuals for their children. “*To me, a mother means. infinite selflessness and affection for the child. It’s like a burning candle that keeps melting*” (Participant 6).

#### 3.2.2 Transformation in life

Pregnancy and motherhood in adolescence brought significant changes to the participants’ daily lives. These changes started from becoming aware of the pregnancy and encompassed psychological, relational, and other dimensions of life. This category included subcategories such as “psychological transformation,” “diversity and enthusiasm,” “changes in relationships,” “losses,” and “experienced physical problems.”

The participants expressed feelings of empowerment, capability, patience, courage, decisiveness, self-confidence, happiness, increased blessings in life, self-worth, a sense of maturity, growth, and pride after the birth of their child. *“I feel like my life turned around. I think I’m not the same person as before. I feel like I have become more mature,”* said participant number 5.

The participants experienced significant changes in their relationships with others after the birth of their child. They mentioned improved relationships with their spouses and families, reduced conflicts in life, and less interference from the spouse’s family in their private lives.

Another transformation that occurred in the relationships of adolescent mothers was a change in their relationships with friends. Almost all participants reported that their contact with their school friends and unmarried friends was limited. They preferred to create a new form of friendship and connection with other adolescent mothers.

#### 3.2.3 Continuum of support

Adolescent mothers reported the need for support from their surroundings to succeed in their maternal role. This category described the level of the support received by adolescent mothers throughout the motherhood process. On one side, it portrayed sufficient and appropriate support received from the spouse, spouse’s family, and the adolescent mother’s family. On the other side, it illustrated inadequate support from their surroundings. This category included two subcategories: “perceived sufficient support” and “insufficient support.” Almost all participants mentioned receiving support from their spouse, their own family, and the spouse’s family. These supports were reported as assistance in household chores, childcare, financial support, and emotional support.

The participants described the perceived sufficient support in the following expressions, “*From the very first day after my child’s birth, my husband told me not to worry and that he would help me with everything. Really, my husband’s encouragement reassured me a lot*” (Participant 5). Despite receiving support from their surroundings, some participants mentioned not receiving sufficient support from their spouse and family due to distance and family problems.

#### 3.2.4 Emotional reactions to pregnancy and becoming a mother in adolescence

Adolescent mothers displayed different emotional reactions to pregnancy and becoming a mother in adolescence. These reactions were categorized into two subcategories: “positive emotional reactions” and “negative emotional reactions.” Most mothers reported feelings of happiness toward pregnancy, the first moment of seeing their child, breastfeeding, a genuine sense of motherhood, increased love and affection, feelings of calmness and attachment through breastfeeding, and joy in witnessing the stages of the child’s growth and development.

Some participants reported emotions and reactions such as unhappiness, shock, embarrassment, regret, worry, anxiety, fear, anger, fatigue from life, and dissatisfaction with marriage and becoming a mother at a young age. “Sometimes, when she cried a lot and didn’t calm down, I would complain to God and regret it,” said participant number 8.

#### 3.2.5 Adolescent mother vs. adult mother

This category describes the differences and abilities of adolescent mothers compared to adult mothers and included three subcategories: “better understanding of the child by the adolescent mother,” “greater enthusiasm of the adolescent mother,” and, “higher experience of the adult mother.”

Some of the participants mentioned that in comparison to adult mothers, they could better understand their children due to having a smaller age gap. They believed that they would be able to comprehend their children better in the future. One of the participants said, “I think I can understand my children better, but an adult mother might not be able to grasp it” (Participant 2).

Most adolescent mothers participating in this study stated that adult mothers were more experienced, knowledgeable, and skillful in child care and parenting compared to themselves. “*I think they* (older mothers) have more experience than me and have better abilities and more information about child care and upbringing,” said participant number 6.

#### 3.2.6 Concept obtained from the fieldwork phase

Motherhood is a transformative concept in life accompanied by a good sense of being a mother as an angel that elicits positive and negative emotional reactions in the mothers. The types of emotional reaction depend largely on the perceived support from the mother, whether it is sufficient. On the one hand, adolescent mothers considered themselves superior to adult mothers in terms of understanding their children, but they acknowledged the greater experience of adult mothers. Overall, the formation of positive or negative maternal feelings in adolescents was highly dependent on their perceived support.

### 3.3 Summary (combination of fieldwork and theoretical phases findings)

**FIGURE 2:**
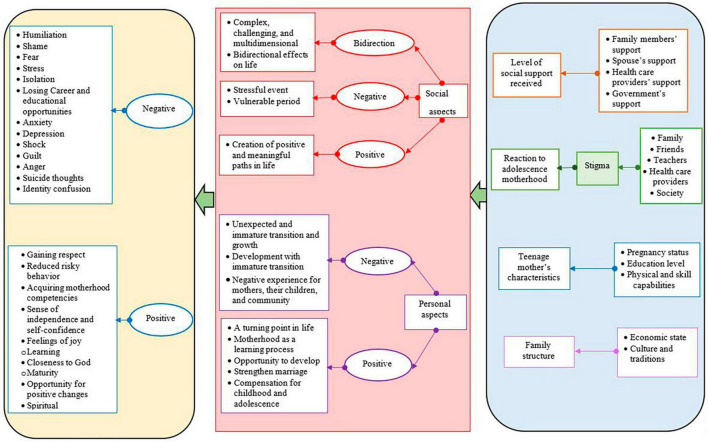
Combination of fieldwork and theoretical phases findings.

Overall, the phenomenon of adolescent motherhood has both positive and negative social and individual dimensions. This concept is influenced by the social support received from the spouse, family, and healthcare providers. The reactions of family members, peers, and society, as well as the individual characteristics of the adolescent and the family structure play, a significant role in shaping the concept of motherhood. Depending on the formation of the maternal concept, positive or negative consequences arise in adolescents. Therefore, it can be concluded that the concept of motherhood among adolescents is a challenging, developmental, multidimensional, and complex process influenced by the level of social support received, reactions to adolescent motherhood, teenage mother’s characteristics, and family structure, which can result in positive or negative psychological consequences and the loss of educational and career opportunities. In Iranian adolescents, pregnancy occurs through formal and culturally/traditionally valued marriage at a young age and this issue can bring sufficient family and social support that leads to spiritual empowerment and positive changes in adolescent mothers (Table 3 and Figure 2).

**TABLE 3:** Combination of fieldwork and theoretical phases findings.

Social aspects	Transformation in life	Changes in relationships
		Creating losses in life
	Continuum of support	Perceived sufficient support, Insufficient support
	Complex, challenging, and multidimensional	Stressful event
		Vulnerable period
		Bidirectional effects on life
		Creation of positive and meaningful paths in life
Personal aspects	Mother as an angel	Responsibility and patience in hardships,
		Love and kindness,
		Selflessness and sacrifice, Caretaker and sanctuary
	Psychological transformation	Diversity and enthusiasm,
		Emotional reactions to pregnancy and becoming a mother in adolescence,
		Enthusiasm and understanding of adolescent mother vs. experience of adult mother
	Unexpected and immature transition and growth	Development with immature transition,
		Negative experience for mothers, their children, and community
	A turning point in life	Opportunity to develop, strengthen marriage,
		Compensation for childhood and adolescence deprivations,
		Motherhood as a learning process

## 4 Discussion

Numerous studies suggest that the transition to motherhood is more challenging for adolescents. Despite international studies, in Iran, the concept of motherhood in teenagers has been paid less attention. Therefore, this research was conducted with the aim of gaining a deep understanding of the concept of motherhood in Iranian adolescent mothers. To clarify this concept, a hybrid model of concept analysis was used. From the reviewed texts, seven attributes were identified for the concept of motherhood: “a process-oriented phenomenon in nature,” “complex, challenging, and multidimensional,” “development with immature transition,” “a turning point in life,” “stressful event,” “affected by several factors,” and “bidirectional effects on life.” Additionally, analysis of participants’ responses in the fieldwork phase revealed five categories for the concept of motherhood in adolescent mothers: “mother as an angel,” “transformation in life,” “attachment support,” “emotional reactions to pregnancy and becoming a mother in adolescence,” and “adolescent mother vs. adult mother.”

The first three attributes of the concept of motherhood in the theoretical phase were “a process-oriented phenomenon in nature,” “complex, challenging, and multidimensional,” and “development with immature transition,” which were not observed in the fieldwork phase. The process of growth and development occurs over a period of time (Fouquier, 2011). Response to physical changes of puberty (Patton and Viner, 2007), development of cognitive abilities, and emotional and behavioral regulation influence this process. Even regarding the physical and developmental effects of pregnancy in adolescence (Steinberg, 2005), some studies attributed the maternal risks associated with pregnancy and childbearing in adolescents more to unfavorable social and economic factors than to chronological age (Kirchengast, 2016). Therefore, it seems that the effects of social and economic factors outweigh those related to physical growth and chronological age (Dzotsi et al., 2020; Santos et al., 2021).

Adolescence is a stage of transition and preparation for moving from childhood to adulthood (Kaye, 2008). Important experiences are acquired in the process of growth and development during this period. The initiation of pregnancy is also a significant transitional period during which women experience several multidimensional changes in their lives. Pregnancy and motherhood represent a critical period of social and psychological stress, reflecting the crisis of maternal identity formation (Maputle, 2012). The results of some studies have shown that becoming a mother in adolescence is an unexpected and immature transition and growth process (Dzotsi et al., 2020; Santos et al., 2021).

“A turning point in life” was another attribute of the concept of motherhood in the theoretical phase. Motherhood is considered a turning point in the life of an adolescent that can bring about fundamental transformations. In a study by Diamond et al., adolescents described their own pregnancy and becoming parents as a turning point in life, as a source of power, coping, accepting responsibility, and an opportunity for positive change. Research also indicates that motherhood during adolescence is regarded as the “spine” and a turning point in life that elevates an individual from a state described as ‘irresponsible’ to a higher state (Clemmens, 2003; SmithBattle, 2005).

The second attribute discovered in the fieldwork phase, i.e., “transformation in life,” aligned with this theoretical attribute. The participants in the study reported experiencing purposefulness and a sense of meaning in life through the birth of their child.

Other attributes of the concept of motherhood were “a stressful event” and “bidirectional effects of motherhood on life.” The significant physical, cognitive, psychological, and social changes that occur during pregnancy and early motherhood have led many to view transition to motherhood as a stress-inducing factor (Schachman, 2001). On the other hand, adolescent childbearing has been a subject of debate as a “crisis” with the perspective that adolescent mothers experience negative consequences in terms of health, childbirth, economic, and social aspects (Govender et al., 2020). In a study by Boyer and Spinney (2016) adolescent mothers considered motherhood a positive yet difficult experience. Similarly, in a study by Rafii et al. (2020) many adolescent mothers experienced conflicting emotions such as regret, confusion, sweet moments, and a sense of pleasure. The fourth attribute in the fieldwork phase, i.e., “emotional reactions to pregnancy and becoming a mother in adolescence,” was consistent with these theoretical attributes.

The participants described motherhood during adolescence as difficult and challenging. They portrayed the mother as a person that endures hardships and sufferings for the sake of her child. Some also highlighted its positive effects, such as increased enthusiasm, happiness, reduced feelings of longing, and a sense of purpose and meaningfulness in life through the birth of their child.

Adults and adolescents have different psychological characteristics, such as avoiding harm, lower levels of behavioral control, increased risk-taking, and a disconnect from traditions, values, and ethics (Moridi and Aminshokravi, 2018). These differences may influence their perception of motherhood. Therefore, the ambivalence felt by adolescents toward becoming mothers may differ from that of adults.

Three antecedents of the concept in the theoretical phase were “the level of received social support,” “reactions of the family, friends, and community members to teenage motherhood,” and “family structure.” The third attribute found in the fieldwork phase, “attachment support,” validated these antecedents in the theoretical phase. The majority of participants stated that they received support in the form of help with household chores and childcare, financial support, and emotional support from their spouse, their own family, and their spouse’s family. However, some mentioned that they did not receive sufficient support from their spouse and family due to distance and becoming a mother. In several studies, the level of support received by adolescent mothers throughout the motherhood process falls into a continuum. On one side of the continuum, there is perceived sufficient and appropriate support from individuals such as the spouse, spouse’s family, and adolescent mother’s family, while on the other side, there is insufficient support from others.

In a study by Govender et al., adolescent mothers mentioned that disclosing their pregnancy had both positive and, in most cases, negative effects on their relationships with their spouses and families. Negative reactions included rejection, anger, and dysfunctional family relationships. Some adolescent mothers stated that their mothers were initially angry but eventually provided support throughout the pregnancy (Govender et al., 2020). The results of a study by Moridi and Aminshokravi (2018) showed that adolescent mothers might feel lonely and abandoned by their friends and family. Joining a group creates a sense of acceptance, socialization, and stability. Therefore, they need more opportunities to form new friendships with other adolescent mothers who share their experiences of being parents and serve as an important source of support and reassurance (Moridi and Aminshokravi, 2018). In a study by Recto and Champion (2020) adolescent mothers received emotional support from various informal sources, including family members such as grandmothers, aunts, and sisters. They mentioned receiving tangible assistance such as infant care, financial support, and provision of supplies for the baby. On the other hand, some others expressed that parents were the source of their emotional distress and increased tension in their relationships (Recto and Champion, 2020). In a study by Santos et al., 2018 the participants emphasized the necessity of family support for adolescent mothers to learn to take care of their children. The results largely depend on the social-cultural context and the perceived level of support by mothers. In Iran, considering the religious and cultural context of the country, pregnancy and childbearing are considered sacred and valuable events. Therefore, families provide a supportive environment for pregnant women, and adolescent mothers benefit from strong family and social support.

As for the reaction of the family, friends, and the community to teenage motherhood, the most common reaction observed in the theoretical phase was anger. The results of a study showed that teenage mothers were stigmatized by the society and likely experienced violence and rejection from their spouses (Atuyambe et al., 2008). In a study by Govender et al. (2020) teenage mothers expressed that knowledge of their pregnancy had a significant impact on their mental health. The reality of being pregnant during adolescence was often distressing for the mothers accompanied by feelings of guilt, shame, and thoughts of suicide (emotional and psychological distress) (Govender et al., 2020). On the other hand, Mangeli et al. (2018a) found that becoming a mother in adolescence fulfilled the childhood dream of participants to become adults so they proudly took on the role of adulthood. In the fieldwork phase, almost all participants expressed the reactions of their spouses and families in the form of receiving support. The socio-cultural background and perceived level of support can have an impact in this regard. In the Islamic culture of Iran, pregnancies occur within the context of marital relationships and are in accordance with Iranian society. The Iranian culture strongly supports reproduction and holds a high value for motherhood.

“Teenage mother’s characteristics” was another antecedent of the theoretical phase. In a study by Sommer et al. (2019) motherhood for teenage women was as real and powerful as motherhood for women at any age. They all expressed unwavering love for their infants. Sometimes, young mothers had a sense of youthfulness and “inexperience” regarding parenting skills and having more energy compared to older mothers. Many of them were happy to share more years of their lives with their children compared to older mothers (Sommer et al., 2019). In another study, one of the main challenges faced by teenage mothers was inefficiency. According to the results of this study, teenage mothers had limited parenting skills such as prenatal care, breastfeeding, and child care (Mangeli et al., 2017). The first and fifth attributes discovered in the fieldwork phase, i.e., “mother as an angel” and “teenage mother vs. adult mother” were consistent with this theoretical antecedent. Some of the participants expressed that due to their smaller age difference with their children, they could better understand them in the future compared to older mothers. They felt they had more enthusiasm, joy, and patience in taking care of their child compared to an adult mother. Additionally, most of the teenage mothers stated that adult mothers had more experience, knowledge, and abilities in childcare and parenting compared to themselves.

Positive and negative psychological transformations were among the consequences of the concept of motherhood in the theoretical phase, which were in line with the fourth attribute in the fieldwork phase, i.e., “emotional reactions to pregnancy and becoming a mother in adolescence.” Consistent with our findings, some studies reported that becoming a mother in adolescence might be associated with positive outcomes, such as increased experience, self-awareness (Moridi and Aminshokravi, 2018), feelings of competence, psychological-emotional maturity, happiness, parenting ability (Anwar and Stanistreet, 2015), commitment, responsibility, meaningfulness in life, Greater perceived social support (Ngum Chi Watts et al., 2015), stronger family relationships, and increased self-confidence and self-esteem (Van Zyl et al., 2015; Mangeli et al., 2018a).

In a study by Gharacheh et al. (2020) some participants mentioned that pregnancy and motherhood provided them with an opportunity to compensate for childhood deprivations, including reducing loneliness. They believed that having children would help them have good friends and spouse (Gharacheh et al., 2020). In a study by Moridi and Aminshokravi (2018) the participants also believed that pregnancy improved their marital life and eliminated their loneliness. In another study, the mothers were highly esteemed, gained a high position within their families and communities, and were accepted as important and respected individuals after becoming a mother (Mangeli et al., 2018a).

In the fieldwork phase, most mothers reported feeling happy about their pregnancy. They also mentioned an improvement in their relationship with their spouses and families and a decrease in marital conflicts with the birth of their child, and expressed that their husbands’ attention to their lives had increased. They felt that their life was not monotonous anymore after the birth of their child and became meaningful and purposeful (Mangeli et al., 2018a). Similarly, in a study by Nor et al. (2019) adolescent mothers found peace and tranquility by surrendering to the will of God to cope with the challenges of pregnancy. According to the participants’ experience, seeking peace from God was related to inner tranquility. They achieved this by performing prayers and regular recitation of the Quran, which also acted as a coping mechanism for maintaining their mental wellbeing. They considered their pregnancy as a spiritual journey that brought them closer to God and viewed it as a positive life experience (Zainudin, 2019). It seems that these feelings are deeply rooted in culture and religious beliefs where mothers are highly honored.

Furthermore, in the fieldwork phase of the present study, some mothers also expressed reactions such as accepting pregnancy based on religious beliefs and expressing gratitude to God for the health of their child. The majority of participants in the present study had positive emotions when faced with motherhood. In the Iranian religious and cultural context, pregnancy and childbirth are considered sacred and valuable events. Therefore, families provide a supportive environment for pregnant women, leading to appropriate coping with the stress and challenges of pregnancy. This finding is supported by several studies (Higginbottom et al., 2006; McMichael, 2013).

Negative psychological consequences such as feelings of worthlessness, shame, fear, stress, anxiety, depression, shock, guilt, anger, suicidal thoughts, isolation, and identity confusion were observed which were consistent with the findings of the fieldwork phase. The role of the mother during adolescence implies that a teenage girl is faced with parental responsibilities since she has to deal with developmental tasks of puberty such as shaping her identity and initiating sexual relationships. Therefore, adolescents have less opportunity to cope with the physical, emotional, and psychological changes of adolescence before exposure to the changes and challenges of pregnancy (Moridi and Aminshokravi, 2018). In a study by Govender et al. (2020) the reality of being pregnant during adolescence was distressing, and emotions such as guilt, shame, and suicidal thoughts (mental and emotional distress) predominated in the minds of teenage mothers. The results of a study by Tirgari et al. (2020) also indicated that unpleasant emotions experienced by teenage mothers included fear and worry, depression, loneliness and isolation, regret and despair, guilt and shame, and doubt. The results of another study also indicated fear of facing challenges due to being unprepared for motherhood, fear of losing the fetus, and fear of childbirth (Gharacheh et al., 2020). In addition, in a study by Wainaina et al. (2021) some teenage girls expressed how being pregnant or becoming a mother at such a young age deprived them of teenage opportunities, freedom of movement, leisure time, buying things for themselves, and having friends.

Another consequence discovered in the theoretical phase was “loss of educational and career opportunities.” Dzotsi et al. (2020) discussed the inability of teenage mothers to continue their education in school due to their pregnancy. In a study by Gbogbo (2020), becoming a mother in adolescence was challenging for all participants. Most of them wished to return to school or learn a profession to secure a better future for their children (Gbogbo, 2020). This consequence was consistent with the second attribute of the fieldwork phase, i.e., “transformation in life.” Dropping out of school due to maternal responsibilities is a reality that indicates a disruption in life projects such as education and personal growth. Among the reasons for dropping out of school, teenage mothers mentioned pregnancy symptoms such as nausea, weight gain, mood swings, and drowsiness, which are particularly associated with increased commitment to the child, especially during breastfeeding (Santos et al., 2018).

### 4.1 Limitations

The present study had several limitations. First, only articles published in English were included in the theoretical phase. Second, the analysis in the theoretical stage was based on studies conducted in multiple countries, while the fieldwork data were collected from only one country. Finally, the findings of the fieldwork phase, like other qualitative studies, are not generalizable.

## 5 Conclusion

The findings of this study clarified the concept of motherhood in adolescent mothers and demonstrated that this concept was defined as a complex, challenging, and multidimensional process in adolescents. Major physical, psychological, social, and cognitive changes during pregnancy and early motherhood in adolescence have made the transition to motherhood a stressful process with numerous responsibilities. Becoming a teenage mother is affected by emotional, physical, spiritual, cultural, family, social, personal, economic, technological, and ethnic backgrounds, local values, and beliefs. Therefore, this period is considered a vulnerable period and a negative experience for the mothers, their children, and society. Understanding the factors influencing the concept of motherhood can be the first step in developing and improving the quality of care for adolescent mothers and, consequently, enhancing the health of these mothers and their children. Moreover, healthcare providers can identify the educational needs of adolescent mothers and risky behaviors by understanding the different dimensions and attributes of the concept of motherhood from the perspective of these mothers, and implement educational programs to enhance their parenting knowledge and skills, modify their risky behaviors, and consequently improve the health of adolescent mothers and their children. Providing healthcare services for adolescent mothers can also contribute to the health of families and communities.

Further studies are necessary to examine the consequences of early motherhood and strategies to help adolescent mothers overcome these challenges. Additionally, the findings can contribute to the development or revision of models, theories, and tools that have been collected for this purpose.

## Data availability statement

The original contributions presented in the study are included in the article/Supplementary material, further inquiries can be directed to the corresponding author.

## Ethics statement

The study protocol was approved by the Ethics Committee of Tehran University of Medical Sciences (IR.TUMS.MEDICINE.REC.1400.815). Informed consent was obtained from the participants before entering the study and the interviews were recorded with permission. The studies were conducted in accordance with the local legislation and institutional requirements. The participants provided their written informed consent to participate in this study.

## Author contributions

All authors listed have made a substantial, intellectual, and direct contribution to the study work, and approved it for publication.
