# Supplementary material for: Developing the concept of maternal in teenage mothers: a hybrid model
Source: Front Psychol. 2024 Jan 8;14:1246882. doi: 10.3389/fpsyg.2023.1246882 (PMC10801042; doi:10.3389/fpsyg.2023.1246882)
Supplement: Supplementary file 1 [file Table_1.docx]

**Supplementary file 1**. Summary of characteristics of the studies selected for the theoretical phase

| Author, Year, Country | Aim | Attributes | Antecedents | Consequences |
| --- | --- | --- | --- | --- |
| Amod et al., 2019, South Africa | To explore the lived experiences  and perceptions of support amongst school-going mothers | Affected by cultural and social context | Unintended pregnancies; Economic hardship; Family destabilization; Unprotected transactional sexual encounters; Insufficient knowledge of contraceptive use; Negative judgement and ridicule of nurses; Parental/caregiver support; Negative reactions from teachers; Stigma from community members and teachers; Financial and emotional support of church group | Shock; Fear; Uncertainty; Disappointment; Confusion; Anger; Shame; Depression; Feeling of lazy and dumb; Guilty feeling |
| Anwar and Stanistreet, 2014, England | Explore the experiences and future aspirations of teenage mothers | Affected by social status: A symbolic identity and a valued role | The mother identity; Stigma; Social support; Future aspirations related to education, training and employment; Negative image of teenage mothers in society; Being judged unfairly | Disconnecting from previous friends; Creating new social networks with maternal identity; Increasing the sense of maturity and motivation to succeed; The conflict between the role of motherhood and the use of opportunities |
| Apolot et al., 2020, Uganda | To explore the maternal health challenges experienced by adolescents during pregnancy, delivery and the postnatal period | The challenging process with cultural, legal, social, and health service dimensions | Stigma and rejection from their parents;  Inappropriate behaviors and poor attitude of health workers toward pregnant adolescents; Physical abuse and violation of human rights; Lack of legal and cultural protection; Inadequate adolescent friendly services at health facilities; Lack of birth preparedness | Shunning seeking of health services; Inferiority feeling |
| Atuyambe et al., 2005, Uganda | To explore problems that pregnant adolescents face in order to design appropriate policies and interventions. | A period with psychological, social and family challenges | Family and social problems; Abortion; Gender issues; Financial problems; Problems related to health services | Rejection, violence, stigma and despair; Denial of paternal responsibility; Abortion due to desperation; Not being understood by the spouse; Deprivation of gainful employment; Negative attitude of health personnel; Poor advices |
| Calver, 2020, UK | To discover transitions to motherhood, young women’s desire for respectability, responsibility, and moral worth | A negative experience for mothers, their children, and society | Negative stereotypes perspectives of society; Stigma | Shame; Achieving responsibility and respectability through engagement in education and employment; Being a “good mother” |
| Campbell & Hart, 2019, Canada | To explore the experiential accounts of adolescent mothers with infants under a year of age and are living in a rural community | Affected by community context; Complex process | Judgment and Stigma; Negative and positive aspects of rural community; Lack of spouse support; Family support; Professional support in the rural community | Attempted to being a good mother; Feeling of isolation; Being an autonomous mother |
| Chemutai et al., 2020, EasternUganda | To explore the lived experiences of adolescent mothers | Complex, challenging and multidimensional process | Poverty and lack of financial support; Need for family support; Marital status; Peer pressure; Being orphans; Parental pressure; Sexual experimentation | Good feeling; Shameful/bad feeling; Fear of reactions from parents, community and colleagues; Fear of labour pains; Stress; Option of abortion |
| Dehghan-Nayeri and Tajvidi, 2014, Iran | To examine the experience of pregnancy among Iranian adolescents | Influenced by context and culture | Psychological reactions; Physical reactions; Spiritual reactions | Ambivalent feelings towards pregnancy; Fear and worry about childbirth, abortion and how to raise a child alone; Fear of tearing the stomach, and the feeling of something moving in the stomach; The miracle of having a baby in the womb; Reading the Quran for the health of the baby; Belief in God's providence |
| Dhaka & Musese, 2019, Kavango | To understand the experiences, challenges, and current coping strategies of young mothers | Inadequate and ineffective coping process | Poor Relationships; Lack of support; Stigmatization and mockery; Using coping strategies; Lack of social support; Lack of family support; Financial burdens; Family support; Government support | School dropouts; Worry; Stress; Regret; Persistent guilt feeling; Feeling like orphans, mistake; coping |
| Diamand et al., 2019, Israel | To explore the experiences of new orthodox teenage girls who became pregnant out of wedlock and raised their children while still teenagers themselves | A period of turmoil and generate feelings of ostracism | Difficulties in relationship; Parenting; Between a lack of separation and disconnection; Lack of family support; Problems related to social adjustment | Feeling of isolation, ambivalence  loneliness, sense of growth, maturation, guilt, fear, joy, and expectation, identity confusion; Coping; Acceptance of responsibility; An opportunity for positive change |
| Dzotsi et al., 2020, Ghana | To explore the challenges experienced by teenage mothers | The sharp and immature transition from adolescence to adult | Parental neglect; Rejection and neglect by partners; Financial and accommodation problems; Stigma | Strained interpersonal relationships; Lacking educational and job opportunities |
| Erfina et al., 2019, Indonesia | To explore adolescent mothers’ postnatal inpatient experiences and healthcare needs as they moved towards their maternal roles | A period with multidimensional care needs and challenges | Breastfeeding problem; Disempowerment in caring for the baby; Health care encounters;  Not understanding adolescent mothers’ needs; Short interaction with midwives; Lack of skills in child caring; Lack of opportunity for engagement with baby  childbirth pain | Stress; Feelings of sorrow and guilty |
| Erfina et al., 2022, Indonesia | To understand the new motherhood experiences of Indonesian adolescent females living with their extended families | Learning process; Affected by family context | Transfer of knowledge between generations; Family support; Living with extended families; Sharing task to taking care of baby with family member; Spouse support; Psychological support from family; Family’s cultural practice related to child's care | Learning parenting skills; Feeling blessed with extended family |
| Field et al., 2020, South Africa | To explore the barriers and facilitators to service access for adolescents in low-resource settings | Influenced by social, cultural and environmental factors | Different levels of mental health literacy; Need for mental health screening; Confidentiality; Social support; Stigma | Resistance to referral; Positive experiences of counseling |
| Franco-Ramírez et al., 2019, Mexico | To understand the structure and content of the maternal representations of Mexican teenagers during their first pregnancy | A period with premature duties | Unplanned pregnancy; Postponed plans; Conducting encouraged activities for the link with child; Interaction with the family; Spouse support; Lack of spouse support; Parental support; Lack of parental support | Worried about child's health; Happiness for fetal movements; Sadness for hindered plans; Fear of parturition pains; Anger; Family discord; Leaving school and job |
| Gbogbo, 2020, Ghana | To explore the lived experiences of pregnant adolescents and adolescent mothers coping strategies during their transition to motherhood | Challenging transition period without being ready to become a mother | Unplanned pregnancy; Discover pregnancy; Rejection by family and friends; Lack of knowledge on baby care; Social support; Spouse support; Family member’s support; Financial limitations; Stigma | Attempted to abort; Hating yourself; Embarrassment; Attempted to abort; Denial and suicidal thoughts; Mixed feelings; Anxiety; Lack of self-worth and confidence; Lack of employment and educational opportunities; Desire to abandon the baby in the hospital |
| Gharacheh et al., 2020  Iran | To explore the lived experience of pregnancy in adolescent mothers | Withering while blossoming; An opportunity for their development, strengthening of their marriage, and compensation for childhood and adolescence deficiencies periods; Unexpected development period; Development within development; Stressful event | Insufficient life skills; Negative experiences from others in performing maternal tasks; Negative emotions toward pregnancy and motherhood; Multifaceted needs; Insufficient support | Loss of life, employment and educational opportunities; Mixed feeling; Inner struggle about life opportunities and threats; Seeking support; Thirst for love, attention and respect; Seeking to improve interpersonal relationship; Deprivation of enjoying; Overwhelmed in needs; Ambiguity in the necessity of having a child; Ambiguity in doing maternal responsibilities; Ambiguity in achieving academic goals |
| Govender et al., 2020, Africa | To understand the phenomenon of adolescent pregnancy and motherhood from the perspective of adolescent mothers | Complicated and multifaceted process; Require a multidisciplinary approach to healthcare; Parenting with accompanied by anxiety and stress | Unplanned and imposed pregnancy; Partners’ and families’ reaction to the pregnancy; Spouse support; Lack of spouse support; Lack of family support; Additional responsibilities; Financial hardships; Lack of financial and social support; Financial and emotional support of family; Exposed to mockery and judged by the community; Stigma and discrimination Healthcare services problems; Repeat pregnancy | Guilt, shame and suicidal ideation; Loneliness; Disruption in schooling; Anxiety; Stress; Alienated; Angry; Unhappy; Fear of the future; Attaining the roles of a provider and nurturer; Increased financial hardship; Feelings of social isolation and guilt with repeat pregnancy |
| James et al., 2012, South Africa | To describe the experiences of teenage pregnancy among Xhosa families | Emotional turmoil while trying to adjust to pregnancy | Experiencing intense emotions with pregnancy: Breakdown in relationships with other: Experience of the positive relationship with their boyfriends; Stigma of pregnancy | Sadness and despair; Feeling despairing; Oversensitive to their family’s perceived response; Blamed their mothers for neglecting them; Loss of respect |
| Kagawa et al., 2017, Mexico | To identify the opportunities and challenges in attaining the maternal role among women who began childbearing in adolescence | Learning process; A common occurrence | Economic Challenges; Social Support; Learning to Parent; Financial pressures | Impossibility of completing education; Limited job opportunities |
| Kaye, 2008, Uganda | To explore the experiences of adolescent mothers during the period of transition from childhood to parenthood and strategies employed in coping with the stress of pregnancy, motherhood, and parenthood | A period with changing needs and desires; A bumpy road | Seizing opportunities for change (prosperity); Failure to control stress; Failure and disappointment; Adapting to challenges tolerating the abandonment of support | A positive experience with pride and joy; Passion for the role of parent; Using emotion-oriented stress control methods  Anxiety; Loss of self-esteem; Two-way attitude to motherhood; The heavy pressure of shame; Confusion and the need to find love; Difficulty accessing financial, moral and material support from parents or partners; Being stigmatized by health workers |
| Kazal et al., 2021, Rhode Island | To explore minority, adolescent birth and perinatal experiences | Stressful period | Support networks; Family as source of stress; Family as source of support; Lack of knowledge; Labor pain | Stress and anxiety; Low satisfaction of birth; Advice for peers and health care provider |
| Klingberg-Allvin et al., 2008, Vietnam | To explore the married adolescents’ perceptions and experiences related to transition into motherhood and their encounter with health care service | Dealing with ambivalent feelings and uncertainty | Ambivalent feelings towards motherhood; Being subject to others; Neglect and lack of support from health care providers | Feeling happy and proud; Feeling childish and lacking confidence to cope with motherhood; Trying to please her husband  Lacking the power to choose for pregnancy, childbirth and the use of contraceptive methods; Compliance with extended family norms and rules; Giving in to society's expectations; Frustration due to lack of information; Ignoring women's concerns  lack of confidence; Embarrassment and modesty; Feeling weak and vulnerable; Not getting proper care from health care providers due to lack of money |
| Macintosh and Callister, 2015, Provo | To explore the adolescents' experiences of motherhood as becoming part of their personal identity | The process of dramatic changes in life | Confirming the pregnancy; Surprising physical and emotional changes for teenagers | Fear of reporting pregnancy to parents; Having hopes and dreams for the future of the child |
| Malatji et al., 2020, South Africa | To explore the challenges confronting teenage mothers when they re-engaged with secondary school education post-pregnancy | A difficult life event to cope with it | Lack of parental support; Additional responsibilities; Hostile educators; Wrongful accusations | Struggle to balance school work with childcare; Fear of being judged and humiliated |
| Mangeli et al., 2017, Iran | To explore the challenges experienced by adolescent mothers during the transition to motherhood | Multidimensional challenges process | Multiple responsibilities; Physical problems; Insufficient support; Health problems related to pregnancy; Lack of time and energy; Lack of essential cognitive and practical competency for motherhood | Emotional and mental distress; Role conflict; Fear and worry; Regret and frustration; Guilt and shame; Depression; Disruption in relationships of couples |
| Mangeli et al., 2017, Iran | To explore the factors that encourage early marriage and motherhood in Iranian culture | Affected by various factors (personal, social, economic, cultural, spiritual, and technological) | Financial problem; Instability of family; Excessive interest in friendship status; Urging of parents for marriage and motherhood; Encouraged subjective beliefs for marriage and motherhood; Insufficient awareness | Feelings of loneliness, love, respect, independence |
| Mangeli et al., 2018, Iran | To explore Iranian mothers’ experiences of the  outcomes of early motherhood | Beyond a negative experience; Acceleration maturation; Heavy burden of adulthood on the small shoulders of childhood | Physical and mental health problems; Experiencing numerous difficulties | Strengthening of family relationships; Developing a strong identity; Missing opportunities |
| Mangeli et al., 2018, Iran | To explore the experiences of Iranian adolescent mothers about the maternal role | A vulnerable period with special abilities | Financial, emotional, care, educational, legal and political support; Professional services of health care providers; Having a history of child care; Reliance on spirituality | Acceptance of challenges; Positive attitude; Compliance with conditions; Planning and management of conditions; Acquiring maternal competency |
| Margherita et al., 2017 | To explain the experience of motherhood in young mothers who are daughters of teen mothers themselves from the psychodynamic perspective of intergenerational transmission | Loss process; An unexpected suddenly event | Unplanned pregnancy; Becoming suddenly mother | Confused identity and roles; Loss opportunity for self-exploration; Low levels of employment, and high crime rates |
| Moridi et al., 2019, Iran | To explore the experiences of Iranian married female adolescent in confronting pregnancy | Influenced by the cultural, social, political, and religious contexts of every community | Paradox of acceptance; Economic barriers  religious beliefs; Child as a factor for stabilizing the marital life; Interest in parenting; Desire to experience motherhood | Happiness and satisfaction; Feeling of uncertainty and desperation; Sense of shock and regret |
| Moridi et al., 2018, Iran | To explore the perception of response to pregnancy in Iranian adolescent women | An ambivalent perception ranging from positive to negative perceptions; Fundamental change in life | Unable to perform mothering roles; Stigma | Social isolation; Fear of pregnancy outcomes; Feeling of independence and confidence; Improvement in marital life; Lack of loneliness |
| Naidoo et al., 2021, South Africa | To explore the experiences of schooling and motherhood at a rural secondary school | Affected by social context  Shameful and stressful process | Stigma; Oppression; Lack of family support; Government inadequate support | Shame; Marginalization; Representations of the identities of the teenage mothers  taking the role of a responsible and committed mother constant anxiety and fear |
| Ngum Chi Watts et al., 2015, Australia | To explore the lived experiences of African Australian young refugee mothers | Positive experience despite the associated challenges | Social support; Family support; Insufficient support from spouse | Sense of maturity; Elevated responsibility |
| Nkwemu, et al., 2019, Lusaka Zambia | To explore the experiences of school-going mothers in Lusaka | A period with multiple challenges | Insufficient support and Mockery from teachers; Stigmatization; Multiple roles; Social insecurity; Forced early marriages; Labelling as deviances | Isolation and rejection and Gossip from classmates; Absent from school; Substance abuse; Staying away from home; Ignore any comments |
| Nor et al., 2019, Malaysia | To understand of how pregnant teenagers make sense of their experience with unwanted pregnancy from a psycho-emotional perspective | Turning point of life; Transformation of self | Babies as a blessing | Being closer to God; Spiritual strengthening |
| Osok et al., 2018, Kenya | To understand depression and mental health care barriers associated with  adolescent pregnancy | Influenced by various social determinants | Social stigma; Lack of basic needs provisions and care, economic problems and poverty; Inability in baby care; Spouse support; Lack of family support; Experiencing domestic violence | Low mood; Anxiety; Stress, Denial of the pregnancy; Feeling insecure for future; Shame; Fear; Feeling defeated; End of education; Look for job; Efforts to abortion; Substance use |
| Recto & Champion, 2020, USA | To examine the types of social support received by pregnant and parenting Mexican-American adolescent mothers | A developmental process requires social and emotional support | Informational, emotional and instrumental support from formal and informal sources; Family support; Family as a source of emotional distress; Health care providers’ support; Financial support; Lack of warmth and empathy from health care providers | Emotional distress; Fear of being judged and humiliated; Hiding their worries; Desperation; Depression |
| Rentschler, 2003, USA | To gain a clearer understanding of teens’ perspectives on pregnancy and parenting | Motherhood is a big responsibility | Unexpected pregnancy changes; Positive and negative changes in relationships with others; The idea of motherhood | Feeling shocked; Emotional, physical, behavioral changes; Educational changes; Changes in roles and responsibilities; Get more respect from school teachers; Losing friends; Being the best mother; Meeting the needs of the child; Accepting the consequences of pregnancy |
| Sámano et al., 2017, Mexico | To explore factors in the individual and family context of teenage girls that can be present with teen pregnancy | Affected by individual and family contexts | Family context before pregnancy; Perceptions of feelings; Diverse family relationships; Families' anger; Sadness; Disappointment; Encouragement of an early marriage | Lonely and indifferent to their parents; Intention of seeking love; Self-perception of being a mother; Fulfillment in spite of the fear and worry; Desire to return to school and to work |
| Santos et al., 2018, Brazil | To understand the trajectory of adolescents regarding the first pregnancy, contemplating realities and perspectives | A process with ambivalent conflicts | Insufficient prevention for pregnancy; Forgetting to regularly use contraceptives; Lack of family support; Difficulties in communication; Financial problem | Fear; Hopelessness; Discouragement; Dropping out of school |
| Santos et al., 2021, Brazil | To identify events and experiences of adolescent mothers relevant to their own care and the care of their children | Learning process; Changing process; Developmental event; Multifaceted event and milestone in life trajectory | New responsibilities; Family support; Spouse support | Learning coping strategies; Dropping out of school; Resuming activities over time; Reducing self-care; Learning in everyday life; Fear; Insecurity; Suffering |
| Seamark and Lings, 2004, UK | To investigate the experiences of teenage mothers in relation to their role as mothers and their expectations of their futures | An opportunity to change and grow | Birth and motherhood; Thinking about motherhood; Positive attitude towards motherhood; Wishes for the future | Giving love and affection to the child; Ambivalent reflections from positive to negative about motherhood; Having more motivation; Planning for further education and career development; Optimistic and realistic attitude to the future |
| SmithBattle et al., 2021, Umbrella review | This umbrella review synthesizes the findings of the qualitative reviews and describes the utility of this evidence for improving clinical practice and policy | Ability to create meaningful and positive ways in life | Social inequities; Stigma | Becoming mature; Reducing risky behavior; Repairing relationships; Reinventing the self |
| Soares and Lopes, 2011, Brazil | To understand the motherhood experiences in adolescence within rural settlements | A point of rupture between adolescence and adult life; Affected by changes in the body, individualistic behavior and thinking about a career | Changes in the personal life; Loss of freedom and increased responsibility | Moral blame; Fragilities and lack of preparedness to cope with circumstances; Difficulty accessing school, lack of opportunities, unstable jobs and difficult family life |
| Sommer et al., 2019 | To further understanding of how second-generation Mexican American adolescent mothers perceive their young motherhood experience | Influenced by ethnicity and cultural context | Basic ethnicity; Lack of communication with their mothers | Worry; Happiness; Feeling of lost youth; Frustrations |
| Sychareun et al., 2018, Lao People’s Democratic Republic | To explore factors contributing to teenage pregnancy and the specific challenges adolescent mothers face in accessing maternal health services | Affected by multi-dimensional determinants of local values, beliefs, and practices | Unplanned pregnancy and abortion; Barriers to access to maternal health services; Being norm of pre-marital sex; Early marriage and pregnancy; Traditional customs and liberal attitudes; Contraceptive non-use; Inaccessibility or avoidance of birth control practices; Socio-cultural norms; Unfriendliness attitudes of healthcare workers; Perceived lack of confidentiality and privacy |  |
| Tirgari et al., 2020, Iran | To explore experiences of teen mothers with stress and stressors of early motherhood | A transition period without unaccompanied, readiness and support; Unarmed combat; Unpleasant stressful process; A period with multiple responsibilities | Incompetence to accept maternal responsibilities; Difficult situations of pregnancy; Unplanned pregnancy; Physical problems; Loss of desirable situations; Failure to perform maternal role; Restrictive laws and the school’s disturbing environment; Get help of others for performing maternal role; Lack of support; Inefficiency and lack of readiness, knowledge, and skill; Lack of physical readiness; Multiple responsibilities | Leaving the school; Fear and worry; Regret and helplessness; Guilty  and ashamed; Depression, Loneliness; Isolation; Wander identity; Limited communication with friends; Conflict between dependence and independence; Physical fatigue; Reduction of comfort |
| Twintoh et al., 2021, Ghana | To explore the childcare practices among teenage mothers | A challenging process requires the support of micro, exo, and macrosystems | Healthy and good child positioning and attachment; Breastfeeding practices; Knowledge benefits of exclusive breastfeeding; Complementary feeding practices; Limited skills in childcare practices Family support; Living in poverty environments | Dependence on parents; Inappropriate sleeping |
| Wainaina et al., 2021, Nairobi | To explore psychosocial challenges and individual strategies for coping with mental stress among pregnant and postpartum adolescents | Complex social and cognitive process; Affected by cultural  Context; Affected all aspects of mother’s life | Parents' encouragement of abortion; Economic hardship; Parents' embarrassment and unnecessary burden; Lack of financial support; Neglect and abandonment by partner; Stigmatization by family, friends, and community; Living in a slum environment | Stress; Being chased from home; Social isolation; Loss of life, employment and educational opportunities; Feelings of shattered dreams; Exposure to various diseases; Taking up other positive and negative habits/hobbies |
